# Comprehensive analysis of mixed neuroendocrine non-neuroendocrine neoplasms (MiNENs): A SEER database analysis of 767 cases

**DOI:** 10.3389/fonc.2022.1007317

**Published:** 2023-01-09

**Authors:** Huixin Song, Sen Yang, Yalu Zhang, Yuze Hua, Jorg Kleeff, Qiaofei Liu, Quan Liao

**Affiliations:** ^1^ Department of General Surgery, State Key Laboratory of Complex Severe and Rare Diseases, Peking Union Medical College Hospital, Chinese Academy of Medical Science and Peking Union Medical College, Beijing, China; ^2^ Department of Gastroenterology, Beijing Friendship Hospital, Capital Medical University, Beijing, China; ^3^ Department of Visceral, Vascular and Endocrine Surgery, Martin-Luther-University Halle-Wittenberg, Halle (Saale), Germany

**Keywords:** surveillance, epidemiology, risk factor, SEER (surveillance epidemiology and end results) database, mixed neuroendocrine non-neuroendocrine neoplasms, MiNEN

## Abstract

**Background:**

Mixed neuroendocrine non-neuroendocrine neoplasm (MiNEN) is an extremely rare entity, consisting of neuroendocrine and non-neuroendocrine components. It can occur in various organs throughout the body, with a rising incidence. Its clinical management is a rapidly growing field of interest; however, large-scale patient cohorts are still missing to guide clinical practice.

**Patients and methods:**

The demographic, clinicopathological, and survival information of all patients diagnosed with MiNEN in the national Surveillance, Epidemiology, and End Results (SEER) program database (2000–2017) were extracted and further analyzed. The information of the patients before and after 2010 was compared to understand the epidemiological changes of MiNEN. The characteristics of MiNEN originating in different organs were compared. The clinical significance of surgical resection for metastatic MiNENs was also analyzed.

**Results:**

A total of 1081 patients were screened, and after applying the exclusion criteria, 767 patients were finally analyzed. There was no obvious sex preference (49.2% vs 50.8%, p>0.05) and the majority of the patients were Caucasians (n=627, 81.7%). A total of 88.3% of the patients were older than 50 years old, and the median age was 60 years. 79.3% of the tumors are located in the distal digestive tract, and 67.7% were grade 3/4. Distant metastasis was presented in 33.9% of the patients at diagnosis. A total of 88% of the patients underwent surgical treatments. The number of patients increased 10-fold between 2000 and 2017. There was no significant difference in sex, race, stage, or surgical treatments among the patients diagnosed before and after 2010. More patients older than 60 years were diagnosed after 2010 (p=0.009). The median survival was 61.0 ± 9.8 months for the whole cohort. After multivariate analysis, older age (>60 years, p<0.01), more advanced stage (p<0.01), grade 3/4 (p<0.01), and non-surgical treatment (p<0.01) were independent risk factors for poorer survival. The appendiceal MiNENs showed the best prognosis. A total of 260 metastatic MiNENs were further analyzed. Only patients with metastatic MiNENs originating from the appendix had a potential benefit from surgical resection, compared to other sites (p=0.05).

**Conclusion:**

This study provides the epidemiological, clinicopathological, and survival information of the largest number of MiNEN patients. Although MiNEN is an extremely rare malignant neoplasm, its incidence increases rapidly. The majority of the patients suffered from advanced-stage disease, which highlights the need for improvement of early detection in the future. The appendix is the most common primary site of MiNEN, and surgical resection for selected metastatic MiNEN originating in the appendix has favorable survival outcomes.

## Introduction

Mixed adeno-neuroendocrine carcinoma (MANEC) was first reported by Oberndorfer et al. in 1907 and classified by the World Health Organization (WHO) in 2010. Before 2010, composite carcinoid was the most commonly used name to describe MANEC. It is an extremely rare neoplasm containing at least 30% of both exocrine and endocrine components (1). It has been reported that MANEC can involve multiple organs, including the gastrointestinal (2), respiratory, and urogenital tract (3, 4). Due to its specific architecture and unspecific clinical manifestations, it is always very difficult to have a definitive diagnosis of MANEC before surgery, and under most circumstances, it was diagnosed after surgical resection by a combination of cytological morphologies and immunohistochemical staining. In the WHO classification (2019), the nomenclature of mixed neuroendocrine non-endocrine neoplasms (MiNENs) was proposed to describe a wider range of these mixed neuroendocrine neoplasms in the digestive system (5, 6).

The majority of the published reports about MiNENs are small retrospective case series, with significant drawbacks in summarizing the clinicopathological characteristics. Recently, a population-level analysis of digestive MiNENs showed a sustained and rapid increase in incidence and mortality from 2010 to 2016 (7). Brathwaite et al. (8) reported a study of the surveillance, epidemiology, and end results (SEER) of appendiceal MiNENs. They found that the overall survival for appendiceal MiNENs was much shorter than of carcinoid tumors but better than that of signet ring cell carcinomas. In addition, several published case series reported that MiNENs in the duodenum (9), gallbladder (10), and liver 11 had extremely poor survival. Similar to other solid neoplasms, radical surgical resection may offer a cure for MiNENs, however, its rapid progression and atypical symptoms in early stages result in a high rate of advanced stages at diagnosis in most patients.

In this study, we utilized the SEER program database from 2000 to 2017 to comprehensively generate evidence for MiNENs patients to better guide clinical practice.

## Patients and methods

### Mining the SEER database

The clinicopathological, surgical, and survival information of the MiNEN patients were extracted from the SEER database (2000–2017) by using the SEER* Stat 8.3.6.1 software. The inclusion criteria were as follows: (1) patients were pathologically diagnosed with MiNENs from January 1, 2000, to December 31, 2017; (2) patients had an active follow-up and did not suffer any other kind of malignant tumor. The exclusion criteria were as follows: (1) patients died from any other cause other than MiNENs; (2) patients whose cause of death was missing or unknown; (3) patients with other malignant tumors.

**Figure 1:**
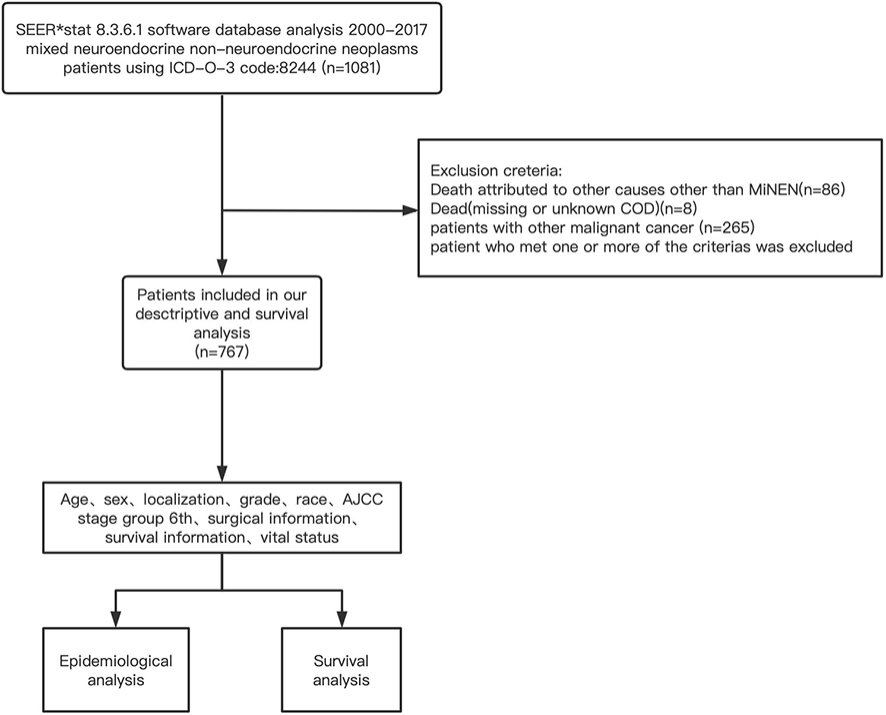
Flow diagram of SEER database mining strategy.

The setting of mining strategies of the SEER database was as follows: “cases in research database”, “year of diagnosis = 2000–2017”, “diagnostic confirmation = microscopically confirmed”, and “ICD-O-3 code:8244”. Details of the data extraction are presented in **Figure 1**.

The following variables were extracted: patient ID, age recorded with exact age and 85+, sex, race recode, year of diagnosis, site according to ICD-O-3/WHO 2008, vital status, survival months, COD to the site, SEER cause-specific death classification, survival months, grade of differentiation, summary stage 2000 (1998+), derived AJCC Stage Group, 6th ed (2004-2015), derived AJCC T, 6th ed (2004-2015), derived AJCC N, 6th ed (2004-2015), derived AJCC M, 6th ed (2004-2015), the reason for no cancer-directed surgery, SEER cause-specific death classification, SEER other cause of death classification, COD to site and the total number of in situ/malignant tumors per patient.

### Statistical analysis

Continuous variables were presented as mean± SD, with analysis using a t-test, analysis of variance (ANOVA), or Mann-Whitney U test. Categorical variables were compared using the Chi-squared test and survival information was analyzed with Kaplan–Meier plotting. The 1-year, 2-year, 3-year, and 5-year mortality ratios were estimated by Kaplan-Meier curves in consideration of sex, age, tumor localization, differentiation stage, AJCC staging group, ethnic origin, surgical information, and summary stage. Univariate and multivariate analysis were performed with a logistic regression model. All tests were two-tailed and a p-value of less than 0.05 was considered statistically significant. The analyses mentioned above were performed using GraphPad Prism 8.2.0 and IBM SPSS statistics 21 (SPSS Statistics V21, IBM Corporation, New York).

### Data availability

Primary data is available online and from the corresponding author (QFL) upon reasonable request.

### End points

The main endpoint was cancer-specific mortality (CSM) according to the data in the SEER database. CSM refers to deaths from MiNENs according to the recorded cause of death. Survival time was the duration from the initial diagnosis to death from cancer or to the last follow-up.

## Results

### General characteristics of the study population

There were 1081 individuals diagnosed with MiNENs in the database from 2000 to 2017, of which 767 were eligible for the study. In the analyzed cohort, 376 (49.2%) were female and 390 (50.8%) were male. The patients enrolled were largely Caucasians (n=627, 81.7%). MiNENs were mainly diagnosed between the ages of 50 and 70 (425, 55.4%), with a median age of 60 years. The number of patients below 50 (n=163, 21.3%) and above 70 (n=179, 23.3%) were similar. Only 3.8% of the tumors occurred in the small intestine, followed by 9.3% in the organs derived of foregut(respiratory, esophagus, stomach, hepato-biliary-pancreatic), 26.4% in the colon-rectum, and 52.9% in the appendix.

Of all included patients, 8.3% of patients (n=64) had well-differentiated tumors, 14.3% of patients (n=110) had moderately differentiated tumors, and 39.6% of patients (n=304) had poorly differentiated tumors. 8% of patients (n=61) had undifferentiated tumors, and the grade information was missing in 29.7% of the patients. Only 49 patients (6.4%) were diagnosed with early-stage of MiNENs. Stage II, III, and IV of the AJCC staging had similar proportions (17.7%, 13.3%, 19.3%). The 6^th^ AJCC staging information was unknown in 332 cases (43.3%). Localized, regional, and distant stage accounted for 22.7%, 38.6% and 33.9% of patients, respectively. Only a small proportion of patients (n=92, 12%) did not receive surgery after being diagnosed with MiNENs. Chemotherapy information was largely missing or incomplete and was therefore not further analyzed. Detailed information was shown in **Table 1**.

**Table 1:** Comparison of demographic and clinical information between patients diagnosed with MiNENs before and after 2010.

Classification	Variables	Total		≤2010		>2010		p-value
		N	%	N	%	N	%	
**Sex**	Female	377	49.2	123.0	51.5	254.0	48.1	0.40
	Male	390	50.8	116.0	48.5	274.0	51.9	
**Age**	<60 years	373	48.6	133.0	55.7	240.0	45.5	0.009
	≥60 years	394	51.4	106.0	44.4	288.0	54.6	
**Race**	White	627	82.1	191.0	80.6	436.0	82.7	0.48
	Non-white	137	17.9	46.0	19.4	91.0	17.3	
**AJCC staging (6^th^)**	I+II	183	42.3	69.0	43.7	114.0	41.5	NA
	III+IV	250	57.7	89.0	56.3	161.0	58.6	
**Differentiation grade**	Grade 1-2	174	32.3	50.0	39.7	124.0	30.0	0.04
	Grade 3-4	365	67.7	76.0	60.3	289.0	70.0	
**Summary stage**	Localized	174	23.8	67.0	29.4	107.0	21.3	0.06
	Regional	296	40.5	87.0	38.2	209.0	41.6	
	Distant	260	35.7	74.0	32.5	186.0	37.1	
**Surgical information**	Surgery performed	675	88.0	217.0	90.8	458.0	86.7	0.11
	No surgery	92	12.0	22.0	9.2	70.0	13.3	

NA, not available.

### Patients diagnosed with MiNENs before 2010 versus after 2010

**Figure 2:**
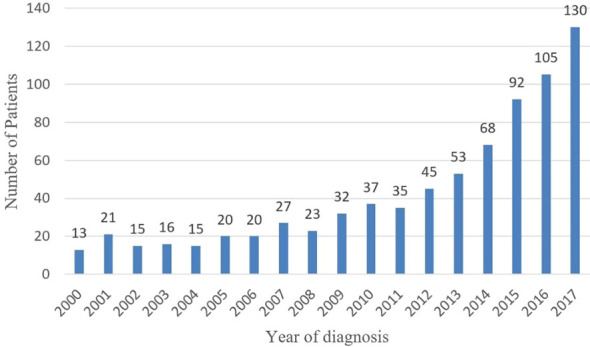
Number of patients diagnosed with MiNENs during 2000-2017.

The number of patients diagnosed with MiNENs significantly increased from 2000 to 2017 (**Figure 2**). To evaluate the effect of the year of diagnosis, we separated the 767 patients into two groups: diagnosed 20 from 00 to 2010(group 1, n=239, 31.2%)and from 2011 to 2017 (group 2, n=528, 68.8%) (**Table 1**). There was no significant difference in sex, race, surgery and summary stage between these two groups. There were more patients over 60 years old in group 2 (54.4%) than in the other group (44.4%) (p= 0.009). Causations took the most part in both groups, with no significant difference (group 1:80.6%, n=191; group2:82.7%, n=436; p=0.5). A higher rate of grade 3/4 MiNENs was observed in group 2 (60.3% vs 70.0%, p=0.04). There was also a lower rate of localized MiNENs in this group (29.4% vs 21.3%, p=0.06).

### Survival analysis for MiNEN patients

**Figure 3:**
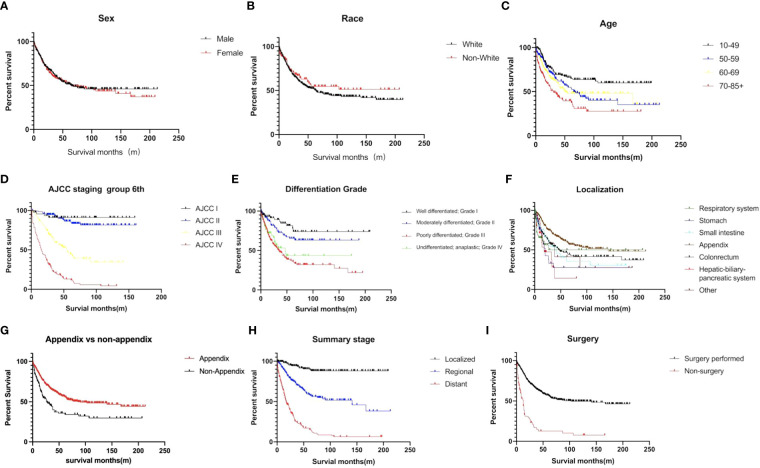
Survival curves of MiNENs depending on **(A)** sex (p>0.05), **(B)** age (p<0.001), **(C)** race (p>0.05), **(D)** AJCC staging group (p < 0.001), **(E)** differentiation (p < 0.001), **(F)** tumor localization (p < 0.001), **(G)** appendix vs non-appendix (p < 0.001), **(H)** summary stage (p < 0.001), and **(I)** surgical information (p < 0.001).

The estimated median survival time of these 767 patients was 61.0 ± 9.8 months. The estimated 1-year, 2-year, 3-year, and 5-year survival rate was, 79.4%, 66.2%, and 58.6%, respectively. The estimated 1-year, 2-year, 3-year, and 5-year survival rate of each variable are presented in **Table 2**. Non-appendical site (p<0.001), Older age (p<0.001), advanced AJCC staging (p<0.0001), grade 3/4 (p<0.0001), summary stage (p<0.0001), non-surgical treatment (p<0.0001) and distant metastasis (p<0.0001) were shown to be independent risk factors for shorter survival (**Figure 3**).The sex and race of the patients did not correlate with survival (**Figure 3**, **Table 3**) Multivariate analysis of factors associated with mortality in MiNEN patients revealed that age, localization, AJCC staging, differentiation, summary stage and surgical choice were independent factors associated with prognosis (**Table 4**).

**Table 2:** Survival regarding different variables.

Classification	Variables	1 year survival rate (%)	2 year survival rate (%)	3 year survival rate (%)	5 year survival rate (%)
**Sex**	Female	79.1	65.3	57.4	51.8
	Male	79.7	67.0	59.6	48.9
**Age**	10-49 years	89.2	76.9	68.9	64.5
	50-59 years	86.7	71.2	63.1	53.1
	60-69 years	76.6	64.8	58.5	NA
	70-85+ years	64.3	51.2	42.7	34.3
**Localization**	Respiratory system	59.2	48.6	NA	NA
	Stomach	68.5	35.8	26.2	NA
	Hepatobiliary and pancreatic system	59.3	NA	14.2	NA
	Small intestine	75.3	62.7	NA	35.7
	Appendix	88.9	75.1	68.9	60.2
	Colorectum	67.4	58.9	49.5	41.7
	Other	71.0	NA	39.9	NA
**Differentiation**	Grade1	91.9	89.9	85.8	80.3
	Grade 2	90.7	81.6	72.6	65.3
	Grade 3	73.7	56.2	46.4	35.1
	Grade 4	66.2	60.9	48.8	39.9
**AJCC staging(6^th^)**	I	95.6	93.4	91.1	91.1
	II	97.8	94.0	92.2	84.3
	III	85.1	70.8	56.3	42.3
	IV	54.7	33.8	19.7	12.1
**Race**	White	79.2	65.8	57.9	50.2
	Non-white	80.3	68.1	61.1	51.8
**Surgical information**	Surgery performed	83.9	71.4	64.2	55.7
	No surgery	44.4	25.3	14.7	11.1
**Summary stage**	Localized	97.5	94.5	92.9	88.8
	Regional	87.7	76.9	69.0	57.0
	Distant	59.8	37.3	23.3	14.2

NA, not available.

**Table 3:** Univariate analyses of risk factors for survival.

Classification	Variables	Median survival(m)	SE(m)	Univariate analysis	
		61.0	9.8	95%CI	p-value
**Sex**	Female	62.0	15.3	32.00-92.00	0.84
	Male	52.0	12.2	32.15-79.86	
**Age**	<60 years	107.0	NA	26.51-49.49	<0.01
	≥60 years	38.0	5.9	41.74-80.26	
**Race**	White	61.0	8.4	44.5-77.5	0.52
	Non-white	103.0	NA	NA	
**Localization**	Appendix	141.0	NA	NA	<0.001
	Colorectum	41.0	13.1	15.4-66.6	
	Small intestine	36.0	8.3	19.8-52.2	
	Others	26.0	6.4	13.4-38.6	
**AJCC staging (6^th^)**	I	NA	NA		<0.001
	II	NA	NA		
	III	54.0	9.5	35.3-72.7	
	IV	14.0	1.7	10.5-17.5	
**Differentiation**	G1-G2	NA	NA	NA	<0.001
	G3-G4	33.0	3.8	25.5-40.5	
**Summary stage**	Localized	NA	NA	NA	<0.001
	Regional	141.0	46.6	49.7-232.3	
	Distant	16.0	1.4	13.2-18.8	
**Surgical information**	Surgery performed	86.0	NA	3.3-14.7	<0.001
	No surgery	9.0	2.9	41.7–80.3	

NA, not available.

**Table 4:** Multivariate analyses of risk factors for survival.

Classification	Variables	HR	Multivariate	
			95%CI	p-value
**Age**	<60 years	1.0	1.1-2.0	0.02
	≥60 years	1.5		
**Localization**	Appendix	1.0		
	Colon-rectum	1.4	0.74-2.5	0.3
	Respiratory/ upper gastrointestinal/hepatobiliary-pancreatic organs	1.6	1.2-2.3	0.004
	Small intestine	1.3	0.77-2.1	0.4
**Differentiation grade**	G1-G2	1.0		
	G3-G4	1.9	1.3-2.7	0.001
**Summary stage**	Localized	1.0		
	Regional	5.8	2.8-12.1	<0.0001
	Distant	16.7	8.0-34.8	<0.001
**Surgical information**	Surgery performed	1.0		
	No surgery	2.7	1.7-4.7	<0.001

### Surgical treatment for metastatic MiNEN patients

**Figure 4:**
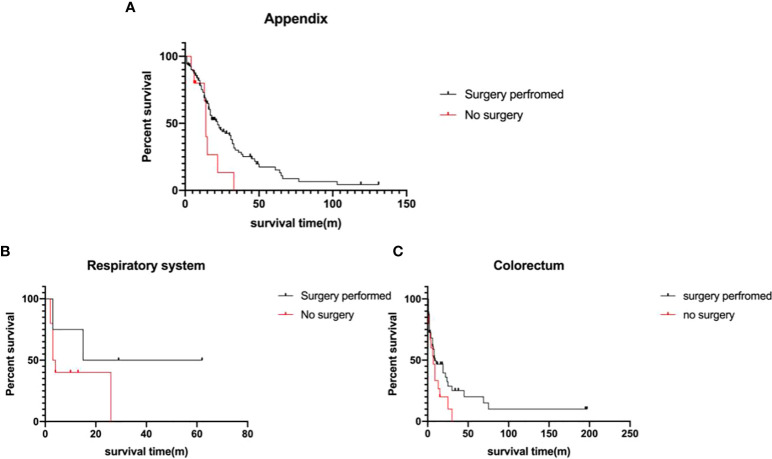
Survival analysis of the metastatic MiNEN Patients **(A)** appendix (p=0.05), **(B)** respiratory system (p>0.05), and **(C)** colorectum(p>0.05), depending on surgical information.

A total of 260 metastatic MiNEN patients were enrolled in this study, 199 of whom (76.5%) underwent surgical resection. The elder patients with metastatic tumors over 60 years old had a lower rate of surgical resection (29.6% vs 16.8%, p=0.02). After 2010, the rate of non-surgical choice in metastatic MiNEN patients was 27.4%, in comparison to 13.5% before 2010 (p=0.02). Surgery significantly improved the survival of the patients with metastatic MiNENs (19.0 ± 2.2 months vs 7.0 ± 2.3 months, p<0.001). Further, metastatic MiNENs in organs derived from the foregut (respiratory, upper gastrointestinal, and hepatobiliary-pancreatic), small intestine, and colon-rectum did not have survival benefit from surgical resection, in contrary, surgical resection improved the survival time of metastatic MiNENs originating from appendix (22.0 ± 3.3 months vs 14.0 ± 0.7 months, p=0.05) (**Figure 4**).

## Discussion

The rarity of MiNENs makes it difficult to get high quality evidence from prospective randomized controlled trials to optimize its clinical care. Therefore, retrospective studies at a population-level are an alternative to provide solid evidence to help guiding the clinical management of this rare malignant neoplasm. As the largest cancer registration program, the SEER database covers approximately 30% of the United States population, which is a powerful resource for retrospective studies at a population- level. Previously, only two SEER analyses for MiNENs were conducted. Brathwaite et al. (8) compared the characteristics of appendiceal MiNEN (n=249), with goblet cell carcinoids (n= 944), signet ring cell carcinoma (n=579), and carcinoid (n=960). It was shown that median overall survival for MiNEN was 6.5 years, which was significantly shorter in comparison to 13.8 years for goblet cell tumors, 39.4 years for carcinoids, but longer in comparison to 2.4 years for signet ring carcinoma. However, in their study, MiNENs originating from other digestive organs was not analyzed. Later, Wang et al. (7) analyzed the epidemiological trend of gastrointestinal MiNENs. In their study, 518 cases of gastrointestinal MiNENs were analyzed, and they found that a sustained and rapid increase both in incidence and mortality of MiNENs from 2000 to 2016. Further, appendiceal MiNENs had better median survival, compared to cecal MiNENs (115 months vs 31 months, p<0.001). The details of MiNENs in other gastrointestinal organs, such as stomach, small intestine, esophagus, hepato-biliary-pancreatic systems, was not analyzed. Herein, we further comprehensive analyzed all of the pathologically diagnosed MiNENs throughout the SEER database from 2000 to 2017, to provide the strongest retrospective evidence to improve the clinical care of this rare entity. In this study, we mainly focused on three issues. The first one was epidemiology differences before and after 2010, when MaNEC was officially named by the WHO. The second one was the differences in clinicopathological characteristics and prognosis of MiNENs originating in different organs. The third one was the clinical significance of surgical treatment for metastatic MiNENs.

Increasing reports of MiNENs have been published during the last 20 years, however, the majority of them were small retrospective studies from singe centers, which likely did not show the real epidemiological trend of all of the diagnosed MiNENs (10–13). Our study showed that in 2000, only 13 cases of MiNEN (composite carcinoid) were found in the SEER database, however, by 2017, it increased ten-fold. The incidence of MiNENs showed a substantially increase during the last 20 years. However, similar to other malignancies, such as thyroid carcinoma, lung cancer, breast cancer (14), pancreatic cystic neoplasm (15–17), which also showed rapid increases in incidence during the last 20 years, it is difficult to say that whether the increasing incidence is attributed to real higher tumor occurrence or to more clinical detections due to the wide use of more accurate imaging modalities.

Although MiNENs can occur throughout the human body, much more reports of MiNENs with larger number of patients in distal digestive tract were published than other organs, indicating its anatomical site preferences (7, 18, 19). In a systematic review of lower digestive tract MiNENs, Grossi et al. (20) reported that 60.3% of them were in the appendix, 29.3% were in the colon, and 10.4% were in the anorectum. In this study, we found the majority of the cases in midgut and hindgut organs, and only 13.1% of them were located in organs derived from the foregut, including the respiratory system, esophagus, stomach, and hepato-biliary-pancreatic system. The most common site was appendix (52.9%), followed by colorectum (26.4%). It is worth noting that neuroendocrine neoplasm is a relative common tumor in appendix which has malignant potential. Brighi et al. (21) reported that tumor size larger than 15.5 mm, G2, and presence of lymphovascular infiltration were predictive factors for nodal metastasis.

MiNENs mainly occurred in patients over 50 years, and there was no race and gender preference. The majority of the MiNENs were grade 3/4, and diagnosed at an advanced stage. It should be stated that in the SEER database, the detail of the grading of the MiNENs was missing. The authors did not know how the tumors were graded exactly. Since there were two components in the tumor, theoretically, both of them should be graded. In the WHO (2019) guidelines and the ENETS guidelines, the grading criteria of GEP-NENs was clearly defined according to the mitotic count and ki-67 index. In the guidelines, it was mentioned that both of the two components in the majority of the MiNENs are poorly differentiated, and the proliferation index for the neuroendocrine components was consistent with other NENs. However, if the differentiation of these two components were not consistent, they should be separately graded (6, 22). A total of 33.9% of the patients presented with metastasis when diagnosed. It is worth noting that only 12% of the patients did not undergo surgical resection, thus, most of the patients with metastatic MiNENs in the SEER database underwent surgical resection. However, it does not mean that in the real world, majority of the metastatic MiNENs were surgically resected; on the contrary, it is possible that many of the patients with metastatic MiNENs who did not undergo surgery died without a definitive pathological diagnosis. Generally, the survival of MiNENs was comparable or worse to adenocarcinoma of the involved organs (8, 13, 23). MiNENs of the appendix showed the best survival, followed by colon-rectum, whereas foregut MiNENs had the poorest survival. Multivariate analysis showed that non-appendiceal site, older age, higher grade of differentiation, advanced stage were independent risk factors for survival.

Due to the substantial improvements of multimodal therapies, several metastatic neoplasms can be treated by surgery. For example, resections have been successful to improve the survival of metastatic colorectal carcinoma and neuroendocrine neoplasms in selected patients (24–26). However, the benefit of surgery for metastatic MiNENs is unknown. In this study, 260 metastatic MiNENs were analyzed. The patients with metastatic MiNENs were younger and had a higher ratio of resection. Generally, surgery substantially improved the survival of these patients (86.0 months vs 9.0 months, p<0.001), however, further analyses showed that only appendiceal MiNENs patients had a survival benefit from surgical resection. Due to the incomplete information of the burden of metastatic lesions in liver and the general status of the patients who underwent surgery or not, there were potential selective biases which may have impact on the interpretation of the results. The patients who underwent surgical resection may have better general status and lower metastatic burden in liver.

Neoadjuvant chemotherapy and adjuvant chemotherapy significantly improve survival of several cancers, and the immunotherapies by targeting immune check points, are successfully used in certain cancers (27–29). In this study, we could not analyze these important aspects of multimodal and novel therapies, as there was not sufficient information available.

The mixed neuroendocrine and non-neuroendocrine components make the biological behaviors of MiNENs more complicated. Genetics analysis have shown that this two components share the same clonal origin (5, 30, 31). However, adjuvant treatments for these two components are largely different (31). In addition, although some molecular targeted drugs and immunotherapies have been preliminary used to treat MiNENs, the evidence of efficacy is limited, and therefore, their roles in MiNENs need further exploration which is beyond the scope of this study (32).

## Limitations of this study

Although this is the largest retrospective study of MiNENs, it has limitations due to its retrospective registration study in nature. Several important information is missing, such as the ki-67 index, mitotic count, immunohistochemical markers, metastatic tumor burden, general status of the patients, adjuvant treatments, et al. In addition, compared to other malignant tumors, such as adenocarcinoma, squamous carcinoma neuroendocrine neoplasm, it can be very difficult to get a definitive diagnosis of MiNEN by fine needle or cord needle biopsy, therefore, in the real world, a large number of metastatic MiNEN patients who did not undergo surgical resection may die without a definitive diagnosis. Although majority of MiNENs are MANECs, there is a very minor proportion of mixed neuroendocrine non-adenocarcinoma neoplasms (e.g., squamous or sarcomatoid phenotypes), which was not included in this study.

## Conclusion

MiNEN is a rare malignant neoplasm with a rising incidence, which can affect multiple organs throughout the body. MiNEN has an anatomical preference in the distal digestive tract, and the appendix is the most common site. MiNEN shows high heterogeneity of aggressiveness: appendiceal MiNEN has the best survival, followed by colon-rectum. The majority of MiNENs are diagnosed at an advanced stage, therefore, early detection should be improved in the future. Generally, distant MiNENs have a dismal prognosis, and surgical treatment only for selected metastatic MiNENs originating in appendix may be feasible. Currently, there is only sparse data on multimodal, treatment options for this disease.

## Data availability statement

The original contributions presented in the study are included in the article/**Supplementary Material**. Further inquiries can be directed to the corresponding authors.

## Author contributions

QFL and QL designed and supervised the study. HS, SY, YZ, YH, and QFL searched and analyzed the data. HS, JK, and QFL interpreted the data and wrote the paper. All authors contributed to the article and approved the submitted version.

## References

[1] Frizziero M Chakrabarty B Nagy B Lamarca A Hubner RA Valle JW. Mixed neuroendocrine non-neuroendocrine neoplasms: A systematic review of a controversial and underestimated diagnosis. J Clin Med (2020) 9:273. doi: 10.3390/jcm9010273 31963850 PMC7019410

[2] Kim KH Lee HJ Lee SH Hwang SH. Mixed adenoneuroendocrine carcinoma in the stomach: a case report with a literature review. Ann Surg Treat Res (2018) 94:270–3. doi: 10.4174/astr.2018.94.5.270 PMC5931938 29732359

[3] Nishimura C Naoe H Hashigo S Tsutsumi H Ishii S Konoe T. Pancreatic metastasis from mixed adenoneuroendocrine carcinoma of the uterine cervix: a case report. Case Rep Oncol (2013) 6:256–62. doi: 10.1159/000351308 PMC3670638 23741220

[4] Raison N McGovern U Hines J Volanis D. Mixed adenoneuroendocrine carcinoma of the urethra. BMJ Case Rep (2019) 12:e227948. doi: 10.1136/bcr-2018-227948 PMC6453422 30902843

[5] Nagtegaal ID Odze RD Klimstra D Paradis V Rugge M Schirmacher P. The 2019 WHO classification of tumours of the digestive system. Histopathology (2020) 76:182–8. doi: 10.1111/his.13975 PMC7003895 31433515

[6] Wu W Chen J Bai C Chi Y Du Y Feng S. The Chinese guidelines for the diagnosis and treatment of pancreatic neuroendocrine neoplasms (2020). J Pancreatology (2021) 4:1–17. doi: 10.1097/JP9.0000000000000064 34102722

[7] Wang J He A Feng Q Hou P Wu J Huang Z. Gastrointestinal mixed adenoneuroendocrine carcinoma: a population level analysis of epidemiological trends. J Transl Med (2020) 18:128. doi: 10.1186/s12967-020-02293-0 32169074 PMC7071749

[8] Brathwaite S Yearsley MM Bekaii-Saab T Wei L Schmidt CR Dillhoff ME. Appendiceal mixed adeno-neuroendocrine carcinoma: A population-based study of the surveillance, epidemiology, and end results registry. Front Oncol (2016) 6:148. doi: 10.3389/fonc.2016.00148 27379210 PMC4904130

[9] Maeda Y Nakahara O Tamaoki Y Saito S Hasuta S Nasu J. [A case of mixed adenoneuroendocrine Carcinoma(MANEC)of the duodenum with rapid liver metastases after pancreatoduodenectomy]. Gan To Kagaku Ryoho (2018) 45:1747–50. 30587733

[10] Zhang Z Zhong D Feng T Yao Y Huang X. Mixed adenoneuroendocrine carcinoma of the gallbladder. J Gastrointest Surg (2022) 26:503–6. doi: 10.1007/s11605-021-05139-2 34668162

[11] Costa AC Cavalcanti CLC Coelho HGB Leao LHA Soares DTC Santa-Cruz F. Rare mixed adenoneuroendocrine carcinoma of the gallbladder: Case report and review of literature. Am J Case Rep (2021) 22:e929511. doi: 10.12659/AJCR.929511 33945521 PMC8105744

[12] Liu S Zhong Z Xiao M Song Y Zhu Y Hu B. Mixed adenoneuroendocrine carcinoma of the hepatic bile duct: a case report and review of the literature. BMC Gastroenterol (2020) 20:399. doi: 10.1186/s12876-020-01550-2 33238879 PMC7691051

[13] Lin J Zhao Y Zhou Y Tian Y He Q Lin J. Comparison of survival and patterns of recurrence in gastric neuroendocrine carcinoma, mixed adenoneuroendocrine carcinoma, and adenocarcinoma. JAMA Netw Open (2021) 4:e2114180. doi: 10.1001/jamanetworkopen.2021.14180 34313744 PMC8317013

[14] Du XL Song L. Breast cancer incidence trends in Asian women aged 20 or older as compared to other ethnic women in the united states from 2000 to 2018 by time period, age and tumor stage. Cancer Epidemiol (2022) 76:102076. doi: 10.1016/j.canep.2021.102076 34861613 PMC8817625

[15] Cong L Wu W Lou W Wang J Gu F Qian J. Gastroenteropancreatic neuroendocrine tumor registry study in China. J Pancreatology (2018) 1:35–8. doi: 10.1097/JP9.0000000000000005

[16] Xiao SY Ye Z. Pancreatic cystic tumors: An update. J Pancreatology (2018) 1:2–18. doi: 10.1097/JP9.0000000000000003

[17] Lee AW Mendoza RA Aman S Hsu R Liu L. Thyroid cancer incidence disparities among ethnic Asian American populations, 1990-2014. Ann Epidemiol (2022) 66:28–36. doi: 10.1016/j.annepidem.2021.11.002 34774744

[18] Tian F Dai MH Jia CW Liu ZW Li BL. Retrospective analysis of seven cases of pancreatic mixed adenoneuroendocrine carcinoma from a high-volume center and review of the literature. BMC Surg (2019) 19:89. doi: 10.1186/s12893-019-0546-0 31296197 PMC6624901

[19] Harada K Sato Y Ikeda H Maylee H Igarashi S Okamura A. Clinicopathologic study of mixed adenoneuroendocrine carcinomas of hepatobiliary organs. Virchows Arch (2012) 460:281–9. doi: 10.1007/s00428-012-1212-4 22358181

[20] Grossi U Bonis A Carrington EV Mazzobel E Santoro GA Cattaneo L. Mixed adenoneuroendocrine carcinoma (MANEC) of the lower gastrointestinal tract: A systematic review with Bayesian hierarchical survival analysis. Eur J Surg Oncol (2021) 47:2893–9. doi: 10.1016/j.ejso.2021.05.021 34052038

[21] Brighi N La Rosa S Rossi G Grillo F Pusceddu S Rinzivillo M. Morphological factors related to nodal metastases in neuroendocrine tumors of the appendix: A multicentric retrospective study. Ann Surg (2020) 271:527–33. doi: 10.1097/SLA.0000000000002939 29995678

[22] de Mestier L Cros J Neuzillet C Hentic O Egal A Muller N. Digestive system mixed neuroendocrine-Non-Neuroendocrine neoplasms. Neuroendocrinology (2017) 105:412–25. doi: 10.1159/000475527 28803232

[23] Wu W Jin G Wang C Miao Y Wang H Lou W. The current surgical treatment of pancreatic cancer in China: A national wide cross-sectional study. J Pancreatology (2019) 2:16–21. doi: 10.1097/JP9.0000000000000012

[24] Liu Q Zhang R Michalski CW Liu B Liao Q Kleeff J. Surgery for synchronous and metachronous single-organ metastasis of pancreatic cancer: a SEER database analysis and systematic literature review. Sci Rep (2020) 10:4444. doi: 10.1038/s41598-020-61487-0 32157155 PMC7064579

[25] Wu W Chen J Bai C Chi Y Du Y Feng S. The Chinese guidelines for the diagnosis and treatment of pancreatic neuroendocrine neoplasms (2020). J Pancreatology (2021) 4:1–17. doi: 10.1097/JP9.0000000000000064 34102722

[26] Arhin ND Shen C Bailey CE Matsuoka LK Hawkins AT Holowatyj AN. Surgical resection and survival outcomes in metastatic young adult colorectal cancer patients. Cancer Med (2021) 10:4269–81. doi: 10.1002/cam4.3940 PMC8267130 34132476

[27] Lee JH Long GV Boyd S Lo S Menzies AM Tembe V. Circulating tumour DNA predicts response to anti-PD1 antibodies in metastatic melanoma. Ann Oncol (2017) 28:1130–6. doi: 10.1093/annonc/mdx026 28327969

[28] Zhang X Cai X Zheng H. Anti-PD1 or anti-PD-L1 antibodies alone or in combination with chemotherapy first-line treatment of advanced non-small cell lung cancer. Thorac Cancer (2022) 13:1104–5. doi: 10.1111/1759-7714.14358 PMC8977173 35199960

[29] Zhang C Li D Xiao B Zhou C Jiang W Tang J. B2M and JAK1/2-mutated MSI-h colorectal carcinomas can benefit from anti-PD-1 therapy. J Immunother (2022) 45:187–93. doi: 10.1097/CJI.0000000000000417 PMC8986629 35343934

[30] Jesinghaus M Konukiewitz B Keller G Kloor M Steiger K Reiche M. Colorectal mixed adenoneuroendocrine carcinomas and neuroendocrine carcinomas are genetically closely related to colorectal adenocarcinomas. Mod Pathol (2017) 30:610–9. doi: 10.1038/modpathol.2016.220 28059096

[31] Jacob A Raj R Allison DB Soares HP Chauhan A. An update on the management of mixed neuroendocrine-non-neuroendocrine neoplasms (MiNEN). Curr Treat Options Oncol (2022) 23:721–35. doi: 10.1007/s11864-022-00968-y 35347561

[32] Ricco B Salati M Reggiani Bonetti L Dominici M Luppi G. PD-1 blockade in deficient mismatch repair mixed adenoneuroendocrine carcinoma of the stomach: new hope for an orphan disease. Tumori (2020) 106:NP57–62. doi: 10.1177/0300891620952845 32878569

